# Supplementary material for: Patterns of Intron Gain and Loss in Fungi
Source: PLoS Biol. 2004 Nov 30;2(12):e422. doi: 10.1371/journal.pbio.0020422 (PMC532390; doi:10.1371/journal.pbio.0020422)
Supplement: Table S1 — Also available at http://genes.mit.edu/NielsenEtAl/. (4.3 MB ZIP). [file pbio.0020422.st001.zip › NielsenEtAl/html/894.html]

AN3661.1.NCU03925.1.MG01033.1.FG08751.1


```
 CLUSTAL W (1.82) Multiple Sequence Alignments - Introns Inserted


Sequence 1: NCU03925.1	412 aa
Sequence 2: MG01033.1	420 aa
Sequence 3: AN3661.1	444 aa
Sequence 4: FG08751.1	407 aa
Alignment Length: 451 aa
Number Identitical Residues: 116 aa
Alignment Score (without introns) 7008


MG01033.1 	MAS-SFHTKEHVIEAQHIREYPRATANSQEDVLQIAVKQYIPKNNPNPQPGDVTIIGAHA
NCU03925.1	MAS-SFIIKEHKVEGQHIREYARATANSQEEVLYLSVKQYMPKSNPNPQPGDLTIIGAHA
FG08751.1 	MSSFPFTIQEHTIEASHIREYARATSHSQDEKLYLHIKQYTPKDNTAPQKGDVTIIGGHA
AN3661.1  	MSTGPFRIVEHTVPGQHIREYPGATANEQEDILSLVVKQYIPLDNPSPQPGDVTILAAHA
          	*:: .*   ** : ..*****. **::.*:: * : :*** * .*. ** **:**:..**

MG01033.1 	NGFPK0ELYEALWEELLTKCTVHGFKIRSIWITDVAWQGQSGILNEGKLGNDP1SWADAE
NCU03925.1	NGFVK0ELYEPLWEDLLRALSARGIRIRSIFIADAAWQGQSGLINESSLGNDP~SWYDHA
FG08751.1 	NGFPK0HG---------KAKVDSSTKTRLAMIVKLTIPSPN-LTHANVLT--A~SWLDYA
AN3661.1  	NGFPK0ELYEPLWEELHARSKENGFRIRSIWMADVAHQGQSSVVNEDILGNDP1SWFDHP
          	*** * .  ..  ..        . : *   :.. :  . ..: : . * ... ** *  

MG01033.1 	RDLLHMVNHFRDSMPRPLVGIGHSFGGAII~TGLSLLHPRLLQSIVLLDPVIGVFS--SA
NCU03925.1	RDLLCVVNALRREMPRPLVGIGHSFGGNTI~ASLSLMHPRLFSSLVLLDPVISKFAKRGP
FG08751.1 	RDILHMINTFRP--PPPIMAMGHSFGANAL~TNVALLHPRIFTSLVLLDPVISHFASTPG
AN3661.1  	RDLLHLVNVKRKEMPRPIVGVGHSMGGAHL2AQLAIIHPRLLHSLILLDPVIQRQTTQLD
          	**:* ::*  * . * *::.:***:*.  : : ::::***:: *::******   :.   

MG01033.1 	SGAT--------MPMTLSSSRRDLWPSRAAAAAAFAKSPFYRAWDPRVLDAWVQHGLRDC
NCU03925.1	SYGF--------LPMKQSAYRRDVWPSRQAAAEGFKKNKFYQTWDPRCLEVLISHGLHQV
FG08751.1 	ASSAG--------PAAASMVRREVWPSRAEAAASFGRSAFYKTWDPRVLQRWIDFGIRNI
AN3661.1  	ASDLGKQKLVIAKTTQSSTYRRDYWPSRKAALDSFKRNPFYQAWDPRVLDRWVRYGLRDL
          	:   ....   : .   *  **: ****  *  .* :. **::**** *:  : .*::: 

MG01033.1 	PTALYPDAPAGS-----------VTLATTKHQECFTYFRTKAQRLAEDGTTLVLDESRLR
NCU03925.1	S----PEKEE-------------VTLTTSKHQECFTYYRPKKQ------------DQPVP
FG08751.1 	P-----GQES-------------VTLTTTKHQEIFTFLRPSWPAYDPQGKD-IIHPEHTP
AN3661.1  	PTSIYPLDESSSNVASNNSAERPVTLRTTLHQEVFTFSRPNYD--GPPGFAVPVNKVTHP
          	.::  .    .:. ::..::. .*** *: *** **: *..       .           

MG01033.1 	DLPLERRGDADQGPFYQPGSSRIYDMLPNVAPSLLYVVGGTSDVSRPEARRDKLARTGVG
NCU03925.1	DLPPDQAVDPN-FQFYRAEAMMTTHFLPHLRPGVLYVFGSTSDVCPPDTREEKLELTGTG
FG08751.1 	DLDTSLNRRWSTYPVYRPEGPNTVARLPNVRPSVLYVFGGKSNVSPLELQDEKMAVTGTG
AN3661.1  	DLDPDH---LGSWPFYRPEPSRIFAQLPHLRPSVLYIFGGTSDMSTPTMIADKLAHTGTG
          	**  .     .   .*:.        **:: *.:**:.*..*::.      :*:  **.*

MG01033.1 	LGGSGGAAAGRVAEYVIEDVGHLVAMDRPGVCAEQAAAWIGPEIER-WRKEELRELEPWW
NCU03925.1	WNGSGGAKAGRVEVRTVEGFGHLVPMEATTRCAEEAAEFIAKDLEKVWRKEDEDFRKVWK
FG08751.1 	VGGSGGEAHGRVKKVVGENNGHLVPMEDPRMCASAAADWIEAELKRWWVDE--RSYEEWS
AN3661.1  	LGGSGGVTAGRVRDVVLKDKGHLVAQEAPVQCAEEASKWLGPELQR-WREEEKMFQDQWS
          	 .****   ***   . :. ****. : .  **. *: ::  :::: * .*.    . * 

MG01033.1 	RKPGPEKCTMNEQLLSLI-PKPGNPKL-------
NCU03925.1	YKEGTEKRVLSKDFLELMGPPPGRKKTGKEEAKL
FG08751.1 	SKSKEDKTTVSDEFQKYIGKPAPRPGKG-TKAKL
AN3661.1  	QKSKIEKVTIDHRWKAHV-PAPVRPKKKDSKSKL
          	 *   :* .:..     :   . .    . .:.
```
